# Flow-Line Evolution, Defect Formation, and Structure–Property Relationships in Aluminum Alloy Forging: A Review

**DOI:** 10.3390/ma19081665

**Published:** 2026-04-21

**Authors:** HaiTao Wang, GuoZheng Quan, Chenghai Pan, Xugang Dong, Jie Zhou

**Affiliations:** 1 Chongqing Key Laboratory of Advanced Mold Intelligent Manufacturing, School of Material Science and Engineering, Chongqing University, Chongqing 400044, China; 2 Chongqing Dajiang Jiexin Forging Co., Ltd., Chongqing 401321, China

**Keywords:** aluminum alloy forging, flow lines, material flow, forging defects, anisotropy, fatigue behavior, preform design, multiphysics simulation, process optimization

## Abstract

Flow lines in aluminum alloy forgings are not merely post-deformation metallographic features; they are integrated indicators of material transport, microstructural evolution, defect susceptibility, and service performance. This review critically examines the mechanisms controlling flow-line evolution, with emphasis on constitutive flow behavior, dynamic recovery and recrystallization, second-phase redistribution, friction, thermal gradients, and die/preform design. It then evaluates how abnormal flow paths promote key defects, including folding/laps, flow-through discontinuities, vortex-like instability, and exposed flow lines, and distinguishes well-established mechanisms from topics that still rely on indirect evidence. Particular attention is given to the effects of flow-line morphology on anisotropy, notch sensitivity, corrosion-assisted damage, and fatigue life in forged aluminum alloys. Current control strategies, including preform optimization, FE-based backward tracing, multiphysics defect indices, frictional heat management, and isothermal forging, are also assessed. The available literature shows that stable contour-following flow lines are essential for the simultaneous control of defect formation, microstructural homogeneity, and durability, while major research needs remain in in situ validation, quantitative defect criteria, and digitally closed-loop process control. This review is therefore framed as a critical narrative synthesis rather than a formal systematic review; emphasis is placed on forging-centered studies that directly relate flow-path evolution to defect formation, anisotropy, fatigue, and process optimization, while evidence transferred from adjacent processes is treated as mechanistic support rather than equivalent proof.

## 1. Introduction

The concept of material flow in metal forming can be traced back to the classical work of Tresca, whose study on the flow of solids laid the theoretical foundation for understanding plastic flow during forging and related deformation processes [1]. In forging metallurgy, this concept is commonly expressed in the form of grain flow, fiber flow, or flow lines, namely the macroscopic record of the deformation path followed by the material during plastic working [2,3,4]. For aluminum alloy forgings, flow lines are not merely geometric traces of shape change; rather, they reflect the combined effects of plastic flow, grain elongation, second-phase redistribution, and deformation history inheritance [3,4,5]. Because they preserve the spatial memory of deformation, flow trajectories provide an essential basis for correlating forging conditions with internal structure and final service behavior [1,4,5].

Flow trajectories are of central importance in aluminum alloy forging because they directly bridge process design and material response. Their evolution is governed by billet geometry, preform design, die cavity configuration, friction conditions, strain rate, and thermal gradients, and therefore reflects whether material transport within the die is smooth, coordinated, and compatible with the target component contour [4,6]. Continuous and well-oriented flow lines usually indicate stable material flow, more uniform strain distribution, and improved die filling, whereas disturbed or discontinuous flow trajectories often reveal local flow incompatibility, abrupt changes in deformation path, or improper material allocation [5,6]. Accordingly, flow-line analysis has become an important criterion in modern forging design, numerical simulation, and process optimization, especially for thin-walled, ribbed, branched, and other geometrically complex forged components [6].

The engineering significance of flow trajectories is ultimately manifested in their strong influence on the performance of forged products. When flow lines are continuous and aligned with the principal loading direction, they can improve load transfer, retard crack propagation, and enhance damage tolerance; in contrast, misoriented or interrupted flow lines tend to intensify microstructural heterogeneity and mechanical anisotropy [2,3,5]. Previous studies have shown that flow-line orientation can markedly affect plastic deformation and fracture behavior even when the differences in yield strength and ultimate tensile strength are relatively limited [2,6]. For example, Qian et al. reported that specimens tested parallel to the flow-line direction exhibited substantially higher plasticity, with a section shrinkage of 57.51%, whereas specimens loaded perpendicular to the flow lines showed much poorer ductility [1,2]. Likewise, Suris et al. demonstrated that forged specimens with grain-flow orientation aligned with the main loading direction exhibited a fatigue life approximately 2.3 times higher than that of transversely oriented counterparts [5]. In aluminum alloy forgings, Shih et al. further showed that the forging-induced microstructure, including grain morphology, grain-boundary characteristics, and second-phase particle distribution, is closely associated with tensile properties, toughness, and corrosion resistance [3,7].

Abnormal flow trajectories may also act as direct precursors to forging defects and premature failure. Once local material flow loses continuity or deviates sharply from the intended contour, defects such as folding/laps, flow-through, broken or exposed flow lines, and other flow-induced discontinuities may develop [4,6]. These defects are not only manifestations of poor macroscopic flow control, but also indicators of irrational stress and temperature distribution, unsuitable die geometry, or improper preform design [1,4,5,6]. In particular, Chan et al. showed that folding is a typical flow-induced defect arising from irrational material flow in forging and that such discontinuities can be systematically analyzed and mitigated through FEM-based process design [6]. Once formed, these abnormal flow regions act as local stress concentrators and metallurgical weak zones, thereby accelerating crack initiation and reducing fatigue resistance and structural reliability [2,5,6,7,8,9,10]. Therefore, flow-line control is not merely a matter of macrostructural appearance, but a key requirement for defect prevention and service safety in forged aluminum components.

Against this background, the present review provides a forging-centered, process–microstructure–property assessment of flow-line evolution in aluminum alloy deformation. Here, “flow trajectories” denotes the evolving material-flow path during forging, whereas “flow lines” denotes its observable macrostructural or metallographic record after deformation. On this basis, the review examines the formation mechanisms of flow lines, analyzes major flow-induced defects, evaluates their implications for mechanical performance and fatigue resistance, and summarizes digital and process-level control strategies. Table 1 defines the terminology used throughout the article and clarifies the scope adopted for the discussion.

To improve methodological transparency, the present article should be read as a critical narrative review rather than a formal systematic review. The literature base was assembled from peer-reviewed studies on aluminum-alloy forging, with priority given to work that explicitly linked flow trajectories or flow lines to one or more of the following aspects: defect formation, microstructural evolution, anisotropy, fatigue behavior, or process optimization. Studies on casting, semi-solid processing, and friction-stir-based routes were retained only when they provided direct mechanistic analogies for instability, interfacial entrainment, or near-surface damage that could reasonably inform forging interpretation. In the revised version, additional representative studies from Europe and Australia on anisotropic crack deflection, small-crack growth, surface-condition effects, and forged-alloy fatigue stability have also been incorporated to improve the international balance of the reference base [11,12,13,14,15,16].

In terms of alloy coverage, the discussion extends beyond the relatively abundant 7xxx literature to representative 2xxx, 5xxx, and 6xxx systems, including 2A14, 2219, 2524, 5A06, 6061, and 6082. This broader framing is important because the dominant manifestation of flow-line effects is alloy-dependent: precipitation-hardenable 2xxx/7xxx systems often show strong coupling among particle redistribution, recrystallization, and anisotropy, whereas 5xxx/6xxx systems more often highlight surface integrity, grain refinement, or forming-uniformity issues. Accordingly, the review differentiates between comparatively mature conclusions supported by multiple forging studies and emerging hypotheses that still rely on indirect or cross-process evidence [17,18,19,20,21,22,23,24,25,26,27,28].

This terminology framework also clarifies why the present article remains forging-centered: neighboring forming or casting phenomena are cited only when they offer useful mechanistic analogies for the interpretation of flow-line evolution in aluminum alloy forging.

## 2. Formation Mechanism of Flow Lines and Microstructural Evolution During Deformation

### 2.1. Dynamic Softening Mechanisms and Constitutive Rheological Behavior

**Figure 1:**
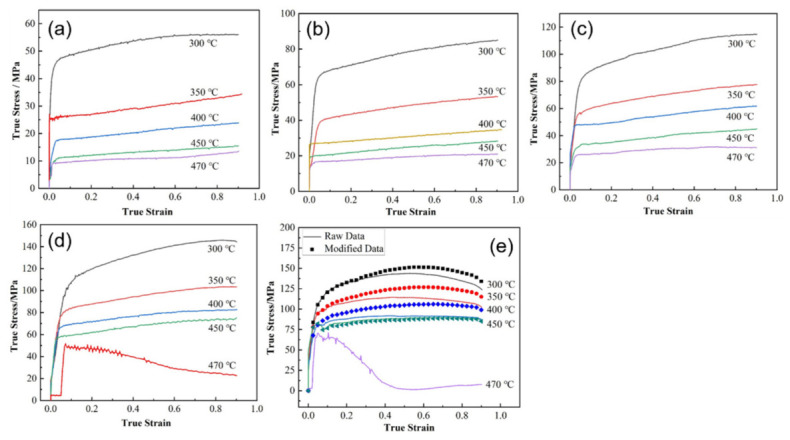
True stress–true strain curves of spray-deposited 7055 aluminum alloy at different temperatures and strain rates during hot compression [13]: (**a**) 0.001 s^−1^; (**b**) 0.01 s^−1^; (**c**) 0.1 s^−1^; (**d**) 1 s^−1^; (**e**) 5 s^−1^. The data for the 5 s^−1^ condition were corrected for deformation-induced temperature rise.

During hot deformation, the rheological behavior of aluminum alloys is governed by the competition between work hardening, arising from dislocation multiplication and interaction, and dynamic softening, induced by recovery and recrystallization. Under elevated temperatures and large strains, dislocations are continuously generated, entangled, rearranged, and annihilated, so that the true stress–true strain curves generally exhibit a rapid initial increase, followed by a peak stress and then a gradual decrease or stabilization once dynamic softening becomes dominant. Such behavior has been widely reported in high-strength 7xxx aluminum alloys, for which the flow stress decreases significantly with increasing deformation temperature and decreasing strain rate [29,30,31,32,33,34]. For representative alloys such as homogenized 7050 and spray-formed 7055, the hot flow behavior can be satisfactorily described by Zener–Hollomon-compensated Arrhenius-type constitutive equations. The reported activation energies are approximately 160.3 kJ/mol for homogenized 7050 and 117.853 kJ/mol for spray-formed 7055, confirming the strong coupling effect of temperature and strain rate on hot deformation resistance [29,35]. As shown in Figure 1, the true stress–true strain curves of spray-deposited 7055 exhibit the typical transition from rapid work hardening to progressive dynamic softening under different temperature and strain-rate conditions, providing a direct macroscopic reflection of the underlying rheological evolution [36].

**Figure 2:**
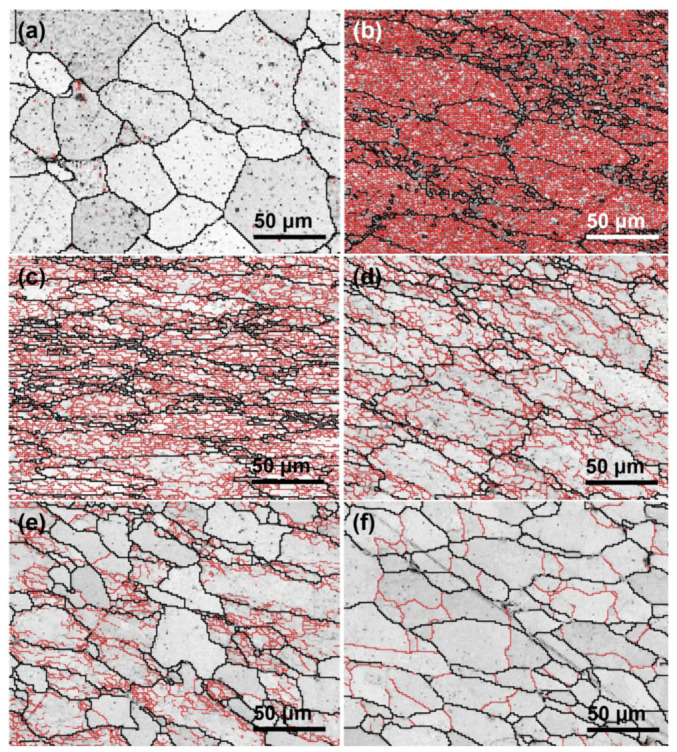
EBSD grain morphology of spray-deposited 7055 aluminum alloy before and after hot compression under different deformation conditions [13]: (**a**) as-deposited; (**b**) 300 °C/5 s^−1^; (**c**) 300 °C/0.001 s^−1^; (**d**) 400 °C/0.1 s^−1^; (**e**) 450 °C/5 s^−1^; and (**f**) 450 °C/0.001 s^−1^. Black lines indicate high-angle grain boundaries (HAGBs, >15°), and red lines indicate low-angle grain boundaries (LAGBs, 2–15°).

**Figure 3:**
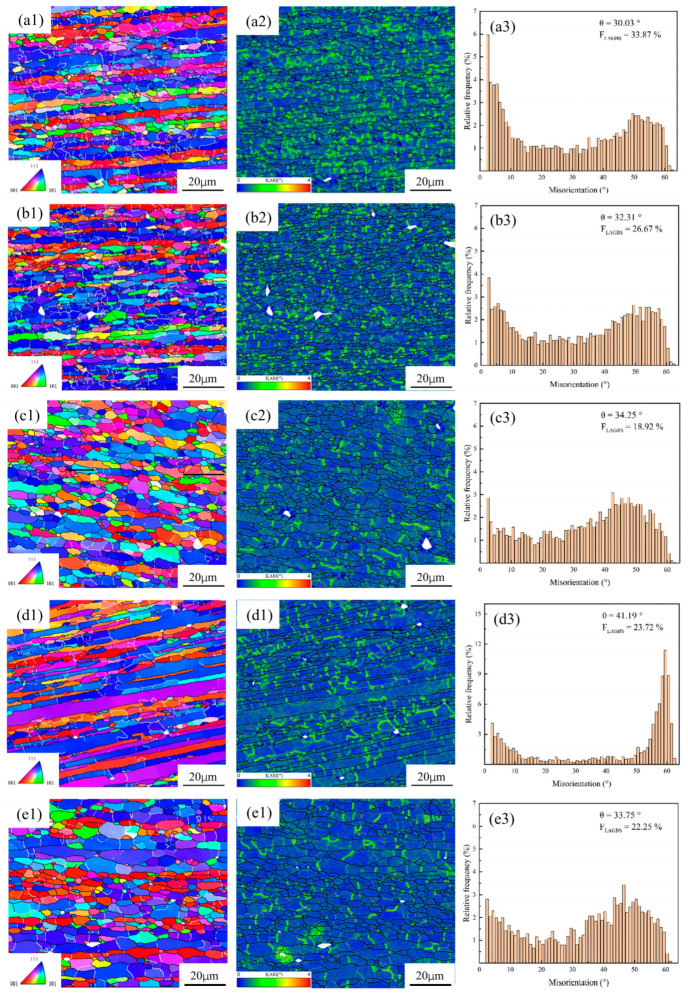
IPF, KAM, and MAD maps of spray-formed 7055 as-forged aluminum alloy deformed at 0.1 s^−1^ under different temperatures [14]: (**a1**–**a3**) 340 °C; (**b1**–**b3**) 370 °C; (**c1**–**c3**) 400 °C; (**d1**–**d3**) 430 °C; and (**e1**–**e3**) 460 °C.

Microstructural characterization by EBSD and TEM further indicates that the dominant dynamic softening mechanism is not controlled by temperature or strain rate alone, but by their combined effect through the Z parameter. In homogenized 7050 aluminum alloy, decreasing Z values lead to a reduction in very low-angle boundaries and a corresponding increase in higher-angle boundaries, indicating progressive subgrain evolution and enhanced softening; under such conditions, dynamic recovery (DRV) remains the primary softening mechanism [29]. In spray-deposited or spray-formed 7055 aluminum alloy, however, the softening behavior is more complex because it is additionally affected by second-phase dissolution and the inherited nonequilibrium microstructure [33,35,36]. At relatively low temperatures and/or high strain rates, DRV dominates and only limited dynamic recrystallization (DRX) occurs. As the deformation temperature rises and the strain rate decreases, the DRX fraction increases markedly. For example, spray-deposited 7055 was reported to undergo nearly complete DRX at 450 °C/0.001 s^−1^ [13]. In spray-formed 7055 as-forged alloy, DRV and continuous dynamic recrystallization (CDRX) dominate at ≤400 °C, deformation substructures and recrystallized grains coexist at 430 °C, and abnormal grain growth appears at 460 °C; moreover, discontinuous dynamic recrystallization (DDRX) is dominant at ≤0.1 s^−1^, whereas CDRX becomes dominant at ≥1 s^−1^ [35]. These trends are clearly illustrated in Figure 2 and Figure 3. Specifically, Figure 2 presents the EBSD grain morphology evolution of spray-deposited 7055 before and after hot compression under different deformation conditions, highlighting the changes in grain morphology and the relative fractions of low-angle and high-angle grain boundaries [36]. Figure 3 further shows the IPF, KAM, and MAD maps of spray-formed 7055 as-forged alloy deformed at 0.1 s^−1^ under different temperatures, revealing the gradual transition from recovery-dominated substructures to recrystallized microstructures and, eventually, abnormal grain growth at higher temperatures [35].

**Figure 4:**
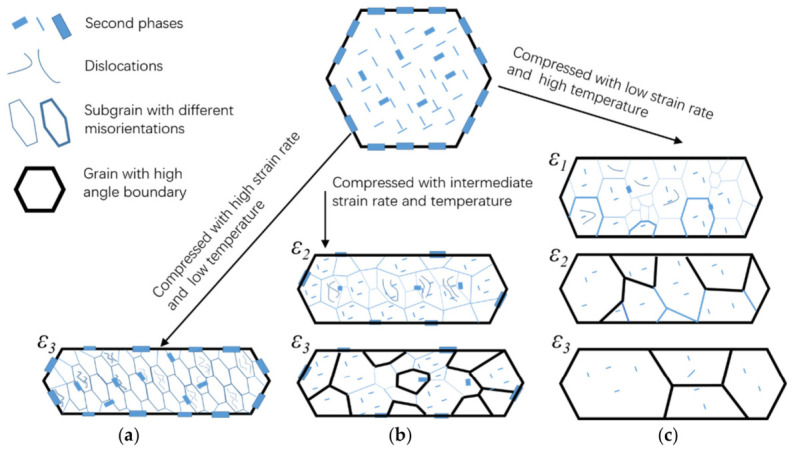
Schematic illustration of the microstructure evolution of spray-deposited 7055 aluminum alloy during hot deformation [13]: (**a**) dynamic recovery accompanied by partial dissolution of second phases; (**b**) dynamic recrystallization via subgrain rotation, subgrain boundary migration, and particle-stimulated nucleation (PSN); and (**c**) dynamic recrystallization associated with homogeneous misorientation increase in subgrains, with a few second phases remaining.

From the perspective of flow trajectory evolution, these dynamic softening transitions are of direct importance because they determine deformation compatibility and strain redistribution during forging. Adequate DRV and DRX can reduce dislocation accumulation, release stored deformation energy, and alleviate strain gradients between neighboring regions, thereby promoting smoother material transport and more continuous flow-line development [29,35,36,37,38,39,40]. By contrast, under relatively high-Z conditions, restricted boundary migration and severe dislocation storage intensify local flow incompatibility and unstable deformation. This tendency becomes particularly pronounced at very high strain rates. In spray-formed 7055 alloy, deformation at 10 s^−1^ produces pronounced dislocation-density gradients within adiabatic shear bands, indicating severe strain localization [35,37,39]. In practical forging, such localized shearing and microstructural discontinuity can be regarded as important precursors to flow-line distortion, shear offsets, and subsequent flow-induced defects. The microstructural basis of these transitions is summarized schematically in Figure 4, which illustrates the evolution from DRV accompanied by partial dissolution of second phases to DRX via subgrain rotation, subgrain boundary migration, particle-stimulated nucleation (PSN), and homogeneous misorientation increase in subgrains [36]. Therefore, constitutive rheology and dynamic softening behavior should be regarded not merely as hot-deformation fundamentals, but as key microstructural foundations for understanding flow-line continuity, flow instability, and defect formation during aluminum alloy forging [29,35].

### 2.2. Redistribution Mechanisms of Second-Phase Particles and the Effect of Multidirectional Forging

In age-hardenable aluminum alloys, alloying elements such as Zn, Mg, Cu, and Si are commonly retained in the as-cast or insufficiently homogenized state as coarse constituent particles, eutectic remnants, or particle-rich networks. During hot deformation, these hard second-phase particles cannot accommodate strain as uniformly as the aluminum matrix. Consequently, severe strain incompatibility develops at particle/matrix interfaces, which promotes particle fragmentation, elongation, spheroidization, and redistribution along the dominant deformation path. In parallel, the deformation zones surrounding coarse particles accumulate high local dislocation density and lattice distortion, thereby facilitating particle-stimulated nucleation (PSN) and local recrystallization. For instance, Robson showed that coarse S-phase particles formed in AA7050 during slow cooling can exceed the critical size required for PSN, while recent work on Al–Zn–Mg–Cu alloys further demonstrated that Al_7_Cu_2_Fe particles can stimulate subgrain formation or recrystallization depending on the deformation condition [41,42]. From the perspective of flow-trajectory evolution, second-phase particles therefore act not merely as passive microstructural constituents, but as active regulators of local strain partitioning, grain subdivision, and flow-line continuity.

The redistribution behavior of coarse particles is strongly influenced by the forging temperature. In both multidirectionally forged 2A14 alloy and forged 2219 Al–Cu alloy, increasing deformation temperature generally promotes particle breakup, spheroidization, and the weakening of particle-rich network features, thereby reducing local stress concentration and improving the metallurgical conditions for subsequent dissolution and precipitation strengthening [17,22]. This temperature-assisted redistribution is therefore important not only for particle-state control, but also for limiting particle-rich flow discontinuities during forging.

Besides temperature, repeated strain-path changes during multidirectional forging play a central role in weakening one-directional particle strings and promoting three-dimensional microstructural homogenization. MDF imposes alternating compression along three orthogonal directions, which continuously reorients the principal stress tensor and disrupts the continuity of particle-rich bands formed under monotonic deformation. In 2A14 alloy, increasing cumulative strain during MDF improves deformation homogeneity, progressively refines the grain structure, and promotes the formation of fine equiaxed recrystallized grains after T6 treatment; the optimum mechanical properties were reported at a cumulative strain of about 3.6 [18]. More recent work on Al–Cu alloys has similarly shown that the metallurgical effect of MDF should be understood as a coupled process of particle fragmentation, particle dissolution, recrystallization, and precipitation redistribution, which ultimately improves three-direction mechanical-property uniformity [19]. Therefore, compared with conventional uniaxial forging, MDF is more effective in transforming strongly aligned particle-rich fiber bands into a more interwoven and spatially uniform flow-line network.

**Figure 5:**
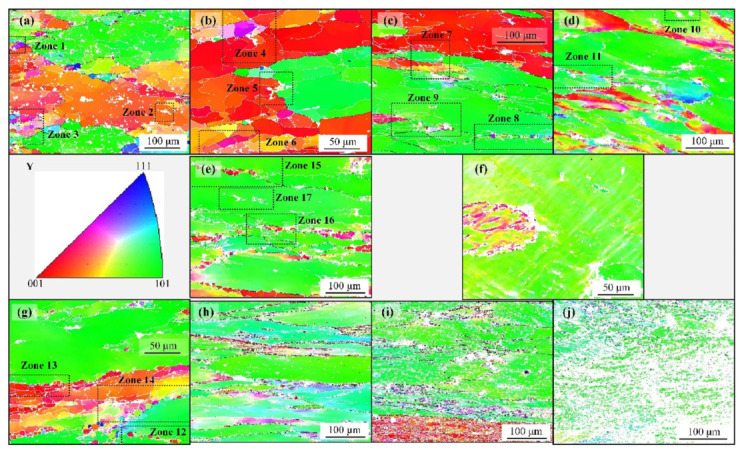
IPF maps of the Al–Zn–Mg–Cu alloy under different deformation conditions [30]: (**a**) 470 °C/0.001 s^−1^; (**b**) 430 °C/0.001 s^−1^; (**c**) 350 °C/0.001 s^−1^; (**d**) 270 °C/0.001 s^−1^; (**e**) 430 °C/0.1 s^−1^; (**f**) 310 °C/0.1 s^−1^; (**g**) 470 °C/0.5 s^−1^; (**h**) 430 °C/0.5 s^−1^; (**i**) 350 °C/0.5 s^−1^; and (**j**) 270 °C/0.5 s^−1^.

**Figure 6:**
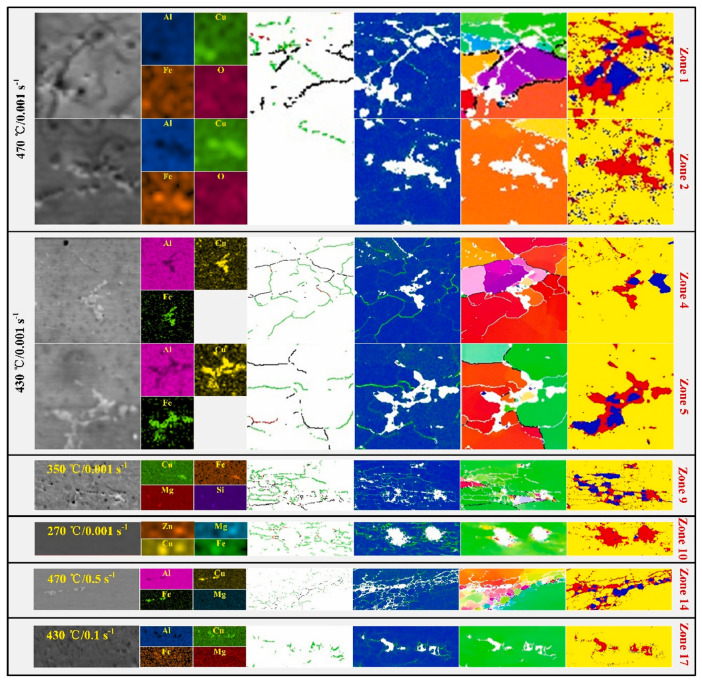
Particle-stimulated nucleation behavior around intermetallic phases in zones 1, 2, 4, 5, 9, 10, 14, and 17 of the Al–Zn–Mg–Cu alloy [30].

The coupling between second-phase evolution and flow-line regulation becomes clearer when local orientation gradients and particle-stimulated nucleation are examined in detail. As shown in Figure 5, the IPF maps under different deformation parameters reveal substantial variations in grain orientation, subgrain development, and recrystallization tendency. More importantly, Figure 6 directly shows that recrystallized grains or subgrains preferentially form around intermetallic particles such as Al_7_Cu_2_Fe, confirming that coarse particles can act as effective sites for local nucleation when the deformation condition is favorable. This observation is consistent with recent cryogenic MDF work on Al–Zn–Mg–Cu alloys, in which increasing the number of forging passes intensified the fragmentation of coarse Fe-rich particles and promoted heterogeneous recrystallization during subsequent annealing [20]. Likewise, improved thermomechanical processing of 2219 alloy has shown that limited particle breakup during hot forging can be followed by much stronger fragmentation during multiaxial cryoforging, thereby increasing particle/matrix interfacial energy and enhancing the driving force for recrystallization and later dissolution during heat treatment [21]. These studies collectively indicate that the key function of MDF is not only strain accumulation, but also the repeated redistribution of particle-controlled deformation zones in three dimensions, which weakens macroscopic anisotropy and improves flow-line continuity.

Overall, the redistribution of second-phase particles and the application of multidirectional forging are intrinsically coupled. Second-phase particles govern local strain incompatibility, recovery, and recrystallization, whereas MDF modifies their fragmentation, dissolution, and spatial redistribution through repeated changes in strain path. Their interaction ultimately determines whether the forged alloy develops continuous and homogeneous flow lines or retains particle-rich discontinuities that may evolve into microstructural weak zones and defect-sensitive regions. Accordingly, second-phase control and strain-path design should be considered simultaneously when optimizing forging routes for high-performance aluminum alloy components [17,18,19,20,21,22,41,42].

## 3. Mechanistic Analysis of Forging Defects Induced by Abnormal Flow Lines

### 3.1. Dynamic Mechanisms of Folding Defects Formation

Folding, or laps, is one of the most frequent and detrimental flow-induced defects in closed-die forging [43,44,45]. It occurs when a surface layer that should remain on the outer contour of the forging is forced to curl, overlap, and become entrapped within the bulk material, thereby creating a sharp geometrical and metallurgical discontinuity. Because the folded interface is not metallurgically bonded in the same manner as the surrounding matrix, it acts as a crack-like defect and can severely deteriorate fatigue resistance, toughness, and surface integrity. Finite-element simulations have shown that folding is not a random surface flaw, but the macroscopic consequence of abnormal free-surface kinematics under non-uniform velocity, stress, friction, and temperature fields during forging [45,46]. Therefore, folding should be understood as a dynamic instability of near-surface flow rather than simply a geometrical imperfection generated at the final stage of die closure.

In practical forging, folding is most likely to occur in regions where the material flow path changes abruptly, such as rib-web transitions, conical passages, sharp fillets, backward-facing cavities, or flange roots. Under such conditions, the inner material, which usually remains hotter and therefore exhibits lower flow stress, tends to move faster than the surface layer adjacent to the die [43,44,47]. By contrast, the surface layer is strongly retarded by interfacial friction and local chilling from the die. This mismatch produces a pronounced velocity gradient across the section, forcing the slower outer material to be overturned, bypassed, or covered by the faster inner stream. Once the free surface folds inward and the two sides of the surface contact each other, a lap is formed. This mechanism has been repeatedly captured by FE analyses in multistation die forging, precision forging, and enclosed-die forging of irregular components. In a classical three-station closed-die operation, the defect was traced to the interaction between blocker design and local flow redirection in the finishing stage, while in an aluminum alloy irregular part with a deep cavity, the lap was directly linked to unfavorable die geometry and the resulting abnormal material transport in enclosed isothermal forging [48,49].

Recent mechanistic studies further indicate that folding defects can be classified according to their dominant flow mode. Gao et al. [48] divided folding in die forging into confluence-type, bending-type, and local-loading-type defects, depending on whether the defect originates from material confluence into a cavity, severe bending and closure of a thin wall, or staged local deformation that generates a surface step and subsequent cavum closure. For complex aluminum alloy forgings, this classification remains useful because different geometric regions within the same component may activate different folding routes, but the common physical origin is still abrupt surface-flow redirection and local flow incompatibility.

Besides the general kinematic classification, die geometry and preform design are decisive in determining whether surface material is guided into the cavity smoothly or forced into self-contact. In enclosed or near-net-shape forging, excessive local material volume, insufficient transition radius, or inappropriate blocker/preform shape can all intensify flow congestion and promote lap formation. Petrov et al. [47] showed that, for an irregular A92618 aluminum alloy forging with a deep central cavity, lap severity was highly sensitive to die geometry, demonstrating that folding can be quantitatively linked to cavity design. Similarly, bevel-gear forging studies have shown that suitable control of billet diameter, punch position, and die configuration can suppress folding while maintaining die filling. Optimization studies based on deformation uniformity or preform sensitivity analysis also confirm that more uniform strain distribution in the preform stage reduces the risk of free-surface inversion and later folding. Accordingly, fold prevention should begin from preform and die-path design rather than from final-stage defect repair.

Interfacial friction is another highly sensitive factor because it controls the velocity contrast between the surface and the interior. When friction is high, the surface layer adheres more strongly to the die, the near-surface flow is retarded, and the tendency for surface overturning or reverse migration increases. Studies on die filling and fold prediction have consistently shown that friction modifies both the free-surface curvature and the local radius ratio of the deformation profile, thereby directly affecting the probability of folding [46,48,49,50]. In counterblow hammer forging, the most realistic die filling was obtained only after careful calibration of the friction factor on different die surfaces, highlighting the strong coupling between friction and cavity filling behavior. Related analyses of surface micro-defects further showed that whether an initial surface irregularity disappears or evolves into a lap depends strongly on defect depth and frictional constraint during upsetting. Thus, friction should be regarded not merely as a load-related parameter, but as a first-order variable governing folding initiation at the free surface.

For aluminum alloy precision forging, thermal conditions further amplify the role of friction because surface chilling and temperature gradients alter local flow stress in a highly non-uniform way. Under approximately isothermal conditions, the deformation-resistance difference between the core and the surface can be reduced, which suppresses excessive velocity mismatch and is therefore beneficial for lap control. Existing experimental–numerical comparisons in irregular aluminum forgings support this interpretation and show that folding can be captured reliably as a flow-driven defect in thermo-mechanically coupled simulation [47].

Overall, the dynamic origin of folding defects can be summarized as follows: abnormal surface kinematics arise when local material flow becomes incompatible with die geometry, preform volume distribution, frictional restraint, and temperature-dependent deformation resistance. Once the free surface experiences inward curvature, step formation, or reverse migration, self-contact becomes possible and a fold is created. Therefore, the mechanistic prevention of folding should focus on suppressing local flow incompatibility by optimizing preform geometry, enlarging critical fillet transitions, reducing redundant material accumulation, controlling friction and thermal gradients, and using FE-based evaluation of free-surface evolution before die manufacture. Recent prediction methods based on folding indices and rapid FE-assisted criteria further indicate that folding risk can be converted from a qualitative post-forging observation into a quantitative design variable during process development.

### 3.2. Physical Metallurgy Mechanisms of Flow-Through Defects

Flow-through defects may be regarded as one of the most detrimental internal flow-line discontinuities in aluminum alloy forgings. In the English literature, this type of defect is more often discussed through related descriptions such as fiber breaking, flow-line interruption, or streamline defects, rather than as a fully standardized term. It usually occurs in regions with abrupt section changes, such as rib–web junctions, deep cavities, and highly localized protrusions, where the originally continuous flow-line network is penetrated or dragged by another high-velocity metal stream. As a result, the parallel primary fiber pattern is locally cut off, deflected, or merged into a new deformation zone, leaving a distinct penetrating band in macro-etching observations and a pronounced structural discontinuity in the forged component [51,52,53].

From the viewpoint of deformation kinetics, the formation of flow-through defects originates from a severe imbalance in deformation resistance between adjacent regions. When a large metal volume is forced to fill a narrow rib cavity or a sharply constricted geometric transition, frictional restraint and local cooling near the die wall markedly increase the deformation resistance of the surface layer, while the hotter inner material retains a much higher flow capacity. Under these conditions, the metal does not deform uniformly; instead, the inner stream tends to seek a lower-resistance path and penetrates across the pre-existing surface-oriented flow network. Zhang et al. showed in isothermal precision forging of complex aluminum-alloy disk structures that excessive redundant metal flowing rapidly in the radial direction at the late stage of die pressing is a direct cause of fibre breaking, which is closely analogous to the flow-through behavior discussed here [39]. Similar FEM-based studies on aluminum forgings also indicate that abnormal streamline transport is strongly associated with nonuniform strain distribution, local stagnation zones, and unfavorable preform geometry [52,53,54].

The metallurgical consequence of such abnormal streamline penetration is not limited to macroscopic fiber disruption. Because the two sides of the penetrated band experience very different strain, strain rate, and thermal histories, they often undergo markedly different levels of recovery and recrystallization. One side may contain highly refined recrystallized grains, whereas the adjacent side may remain coarse or partially recrystallized, thus forming a typical mixed-grain microstructure. For aluminum alloy forgings, this heterogeneity is especially dangerous because coarse-grain regions and low-strain zones are known to correlate strongly with degraded mechanical properties and reduced structural reliability. Weroński et al. showed that coarse-grain defect regions in aluminum forgings coincide with low-strain areas predicted by FEM, highlighting the strong link between deformation nonuniformity and structural discontinuity [52]. More recent work on forged aluminum components likewise confirms that improving deformation-path coordination and suppressing abnormal grain growth are essential for obtaining uniform microstructure and stable performance [23,55,56,57].

**Figure 7:**
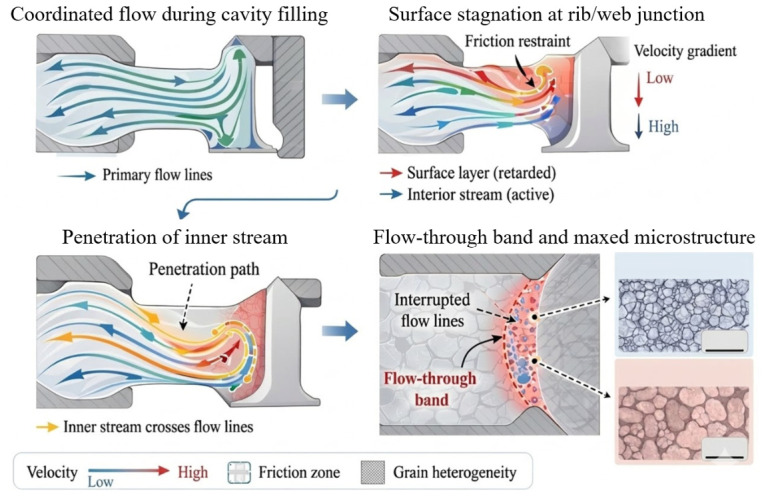
Schematic illustration of the formation mechanism of flow-through defects in aluminum alloy forgings.

As schematically illustrated in Figure 7, the mechanism of flow-through defect formation can be summarized in four steps: first, coordinated flow lines develop during normal cavity filling; second, the surface stream becomes retarded near a narrow transition because of frictional constraint and geometric confinement; third, the hotter inner stream accelerates and penetrates across the slower outer flow-line network; finally, a flow-through band is formed, accompanied by interrupted streamlines and a mixed-grain region after deformation and subsequent heat treatment. For a review article, a redrawn schematic is preferable to direct reproduction of a case-specific figure, because it abstracts the common kinematic and metallurgical features shared by deep-cavity, rib–web, and disk-type forgings [51,52,53].

Overall, flow-through defects should be understood as the combined result of abnormal streamline kinematics and microstructural incompatibility. Their prevention therefore depends on coordinated control of preform shape, material distribution, rib-cavity filling path, friction, and local thermal conditions, so that severe velocity mismatch between the interior and the surface can be avoided. In this sense, the essential strategy for suppressing flow-through is not merely to “repair” the final defect morphology, but to eliminate the underlying flow-path instability that causes streamline penetration in the first place [23,51,52,53,54,55,56,57].

### 3.3. Vortex and Turbulence Instability Phenomena

In conventional closed-die forging of aluminum alloys, material flow is generally expected to remain continuous and ordered, and the flow-line network is usually interpreted within a laminar-like deformation framework. However, under extreme processing conditions, especially in semi-solid die casting, high-pressure die casting, and vortex-assisted friction-stir-based processes, the flow front may lose its stability and develop vortex-like recirculation, swirling streams, or localized high-shear instability. In this context, the term “turbulence” should be used cautiously for aluminum alloy forming; in most cases, flow-front instability and vortex-like recirculation are more accurate than fully developed hydrodynamic turbulence. This distinction is also emphasized by Hu et al., who showed that even when the overall flow remains essentially laminar, local instability at the semi-solid/gas interface can still cause gas entrapment and subsequent defect formation [58].

**Figure 8:**
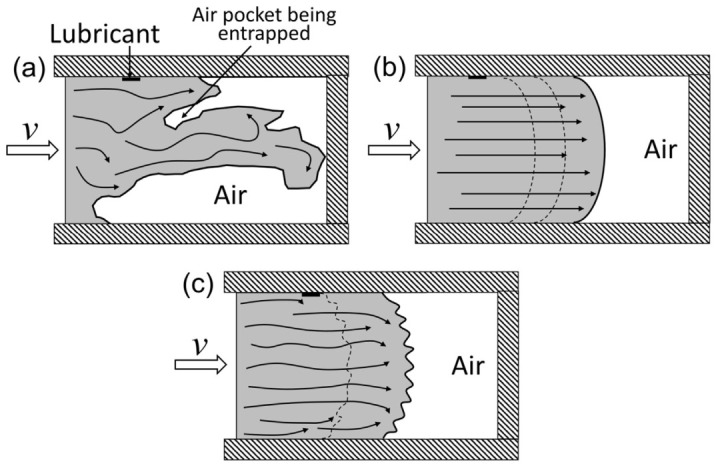
Schematic illustrations of different flow-front states during aluminum die casting [46]: (**a**) non-planar and turbulence-like flow in conventional high-pressure die casting; (**b**) planar and stable laminar flow in semi-solid processing; and (**c**) planar laminar flow with local interface instability at the semi-solid/gas front.

The physical origin of such instability lies in the abrupt redistribution of momentum when a high-speed metal stream enters an expanded cavity, impinges on the opposite wall, and then undergoes separation and backflow. Under these conditions, local recirculating zones may form near the wall or within the flow front, thereby entraining residual air, lubricant decomposition products, or fragmented oxide films into the interior. As shown in Figure 8, the difference between stable planar flow and instability-prone flow-front behavior can be visualized directly: conventional high-pressure die casting is associated with non-planar and turbulence-like flow, whereas semi-solid processing usually shows a more stable planar front; however, once local interface instability develops, gas can still be trapped beneath the advancing surface layer [58]. Vacuum-assisted die-casting studies further indicate that lowering the cavity pressure can suppress entrainment and reduce gas porosity by stabilizing the filling process [59,60].

For aluminum alloy quality, the consequence of vortex-like instability is highly detrimental. The low-pressure core of a recirculating zone can act as an entrainment site for gas and oxide fragments, which later appear as pores, blisters, or bifilm-like discontinuities after solidification or subsequent heat treatment [58,59,60]. In heat-treatable alloys, excessive local thermal–mechanical instability may also accelerate precipitate coarsening and softening. This has been reported in vortex-friction-stir processing of AA6061-T6, where strong vortex-driven material flow is accompanied by significant local microstructural evolution and softening in the thermally affected region [24,61]. Therefore, in aluminum alloy processing, vortex-like instability should be regarded not only as a kinematic abnormality, but also as a precursor to internal defect retention and local property degradation [24,58,61,62].

Overall, vortex and turbulence-like instability phenomena can be understood as special forms of abnormal flow behavior under high kinetic energy, sudden cavity expansion, or intense local shear. Their relevance to forging should therefore be interpreted cautiously: the strongest evidence comes from localized high-velocity regions and from mechanistic analogies with adjacent processes, whereas direct in situ validation in aluminum alloy die forging remains limited. This makes the topic promising, but not yet as mature as folding or flow-through analysis.

### 3.4. Environmental Degradation Effects of Exposed Flow Lines

For aluminum alloy components subjected to long-term cyclic loading, such as aero-engine casings, landing-gear wheels, and high-speed train bearing rings, forged flow lines should remain not only continuous but also conformal to the external contour of the part. Once the streamline network is truncated and exposed at the free surface, a flow-line outcrop or broken streamline defect is formed. In essence, this defect represents the surface termination of a previously continuous forged fiber structure, thereby weakening the microstructural continuity established during forging. Its harmful effect is consistent with the pronounced directional dependence of fatigue behavior observed in forged aluminum alloys, where fatigue resistance is strongly influenced by local forging history, grain orientation, and the alignment between the forged microstructure and the principal service stress direction [63,64].

The formation of exposed flow lines mainly involves two routes. The first is unfavorable metal discharge toward the parting line during die closure. When excessive flash volume or an improper trimming path draws contour-following fibers toward the flash region, subsequent trimming leaves the truncated streamline ends exposed on the lateral surface. The second is excessive machining allowance. Although machining is required for dimensional accuracy, it can directly cut through the forged fiber structure and simultaneously alter the near-surface integrity. For 7075-T6 aluminum alloy, machining-induced changes in surface condition and subsurface integrity have been shown to significantly affect high-cycle fatigue performance, indicating that machining should be regarded not only as a shaping step, but also as a potential source of streamline interruption and fatigue sensitivity [62].

**Figure 9:**
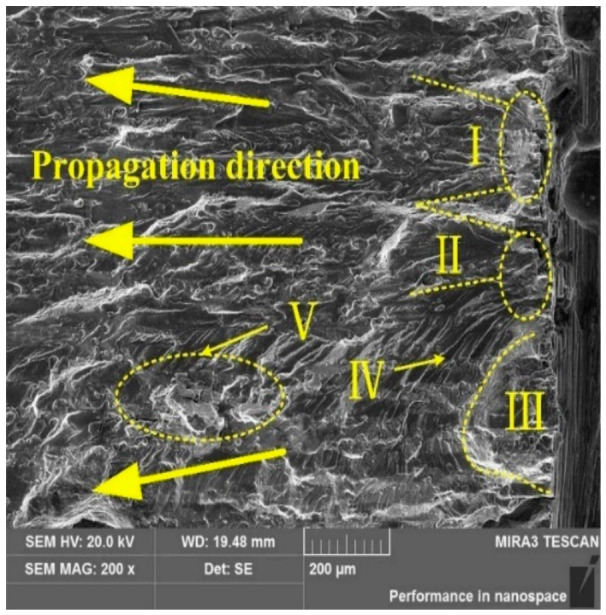
SEM micro-morphologies of the fatigue source area in corrosion-fatigue-tested 2524-T3 aluminum alloy, showing pit-induced crack initiation and subsequent radial crack propagation [54].

The main hazard of exposed flow lines lies in their enhanced environmental sensitivity. Once the truncated streamline ends are directly exposed to a corrosive environment, grain boundaries, dislocation-rich regions, and particle-rich microstructural heterogeneities near the surface can become preferential sites for localized electrochemical attack. In aluminum alloys, corrosion pits frequently act as dominant crack-initiation sites under cyclic loading. For example, corrosion-fatigue tests on 2524-T3 aluminum alloy showed that surface pits can directly trigger crack initiation and promote subsequent crack growth under alternating stress [25]. In high-strength 7xxx alloys, the situation is even more critical because intergranular corrosion and stress corrosion cracking are strongly coupled with grain-boundary precipitates and local electrochemical heterogeneity. Recent work on AA7075 demonstrated that the evolution of η-phase grain-boundary precipitates plays an important role in the interaction between intergranular corrosion and stress corrosion cracking in chloride-containing environments [65]. Therefore, once a flow line is broken and exposed at the surface, the risks of pitting, intergranular attack, and SCC-assisted crack propagation all increase markedly [25,65]. As shown in Figure 9, corrosion pits formed in the surface source region can act directly as crack initiation sites, after which the crack propagates radially into the interior under cyclic loading, illustrating the typical pit-to-crack transition associated with environmentally exposed microstructural discontinuities [25].

From the viewpoint of fatigue failure, exposed flow lines are also detrimental because they destroy the original crack-arresting function of contour-aligned forged fibers. A surface crack initiated at a broken streamline no longer propagates across a continuous and favorably oriented forging structure; instead, it can exploit the truncated boundary network and locally weakened particle-rich regions, thereby accelerating crack advance. This interpretation is consistent with the direction-dependent fatigue behavior reported for die-forged 2014 aluminum alloy aircraft wheels and forged Al–Li 2195 alloy, where local forging path and microstructural orientation strongly influence crack propagation resistance and fatigue life [63,64]. Accordingly, the engineering control of this defect should focus on minimizing unnecessary machining allowance, optimizing flash design and trimming paths, and preserving contour-following flow lines in the final near-surface region, especially for corrosion-fatigue-critical aluminum alloy components [25,62,63,64,65]. For clarity, Table 2 summarizes the major categories of abnormal flow-trajectory-induced defects discussed in Section 3, together with their principal triggers, dominant consequences, and representative prevention strategies.

As summarized in Table 2, these defects differ in their immediate manifestation, but they share a common origin in local flow-path instability and therefore must be mitigated through coordinated preform design, die-geometry optimization, friction control, and thermal regulation.

## 4. Influence of Flow Lines on Mechanical Properties and Fatigue Behavior

From a property-evaluation standpoint, the influence of flow lines is not limited to tensile strength and elongation. In forged aluminum components, flow-path continuity also affects fracture mode, crack-path tortuosity, corrosion-assisted crack initiation, and the stability of fatigue damage accumulation; therefore, this section should be read as a coupled assessment of tensile response, damage tolerance, and durability rather than as a narrow comparison of room-temperature tensile indices alone. This broader interpretation is especially necessary when comparing 2xxx, 6xxx, and 7xxx alloys, because the same macroscopic flow-line disturbance may manifest as directional ductility loss in one alloy system, pit-assisted fatigue initiation in another, and heterogeneous recrystallization or coarse-particle-assisted cracking in a third [3,25,63,64,65,66,67,68,69,70,71,72].

### 4.1. Severe Anisotropy in Strength and Plasticity

Severe anisotropy in strength and, more critically, in plasticity is a common consequence of flow-line-controlled microstructures in high-strength wrought and forged aluminum alloys. During hot deformation, elongated grains, deformation bands, and coarse second-phase particles tend to align along the principal metal-flow direction, thereby forming directional weak paths within the matrix. As a result, the difference in ductility between loading directions is often much larger than the difference in yield strength (YS) or ultimate tensile strength (UTS). This feature is particularly evident in large 7xxx-series forgings, where the most severe loss of ductility commonly occurs along the thickness or short-transverse direction, even when the corresponding strength reduction is relatively limited. For example, in 7085 alloy forgings, the average elongation anisotropy index decreased from 14.0% to 8.6% after temperature-combination-controlled two-stage multi-directional forging, while UTS was maintained without obvious sacrifice. Likewise, multidirectional rotary forging of Al7075 reduced the in-plane anisotropy of both tensile strength and yield strength to about 1%, indicating that path modification can simultaneously improve isotropy and refine the microstructure [26,27,73].

The microstructural origin of this anisotropy lies in the combined effect of flow-line alignment, elongated grain morphology, crystallographic texture, and the directional arrangement of coarse intermetallic particles. When the loading direction is parallel to the streamline network, deformation can be accommodated more uniformly by elongated grains and aligned substructures, and crack propagation is forced to follow a more tortuous path. In contrast, when loading is applied across the flow lines, the continuity of the elongated structure is lost, and microvoids or microcracks can more readily link up along particle-rich interfaces, boundary networks, or locally weakened regions. This tendency is consistent with observations in AA7010-T7452 open-die forgings, where continued forging produced a more fiber-like elongated structure in the L direction and altered the fatigue crack propagation behavior; broken clusters of primary Al_7_Cu_2_Fe particles were observed on fracture surfaces and were identified as relevant damage features in orientation-sensitive cracking. A related trend was also reported for as-rolled 7050 alloy, in which the tensile direction had only a limited effect in the as-quenched state, whereas over-aging made the in-plane anisotropy of elongation much more pronounced because coarse precipitates and visible precipitate-free zones facilitated direction-dependent plastic localization [66,68].

**Figure 10:**
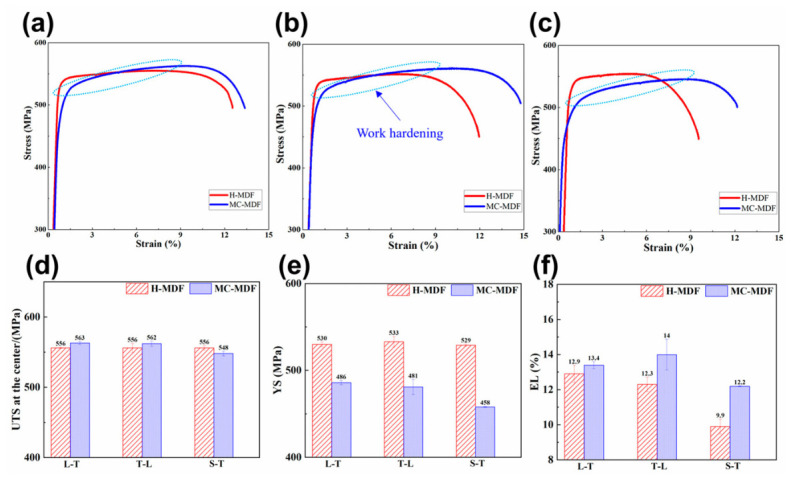
Stress–strain curves and three-direction mechanical properties of 7085 alloy forgings processed by H-MDF and MC-MDF after T74 aging [58]: (**a**) L–T direction; (**b**) T–L direction; (**c**) S–T direction; (**d**) UTS; (**e**) YS; and (**f**) EL.

**Figure 11:**
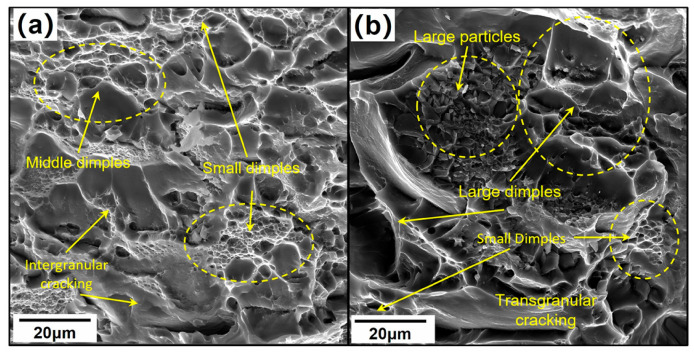
SEM images of tensile fracture surfaces of 7085 alloy forgings after T74 aging: (**a**) H-MDF and (**b**) MC-MDF [58].

**Figure 12:**
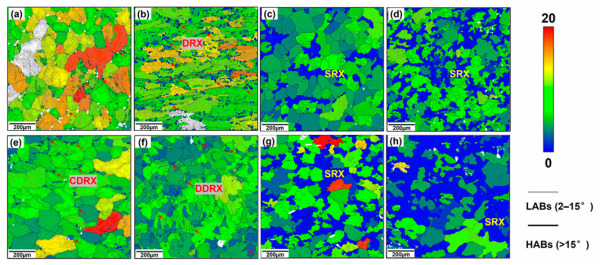
GOS maps and grain-structure evolution of 7085 alloy forgings under different states [58]: (**a**,**c**,**e**,**g**) H-MDF and (**b**,**d**,**f**,**h**) MC-MDF; (**a**,**b**) after the first stage; (**c**,**d**) after inter-annealing; (**e**,**f**) after the second stage; and (**g**,**h**) after solution treatment.

Figure 10 directly compares the tensile response and three-direction mechanical properties of 7085 alloy forgings processed by conventional hot multi-directional forging (H-MDF) and temperature-combination-controlled MDF (MC-MDF). The results show that the directional differences in YS and UTS are moderate, whereas the variation in elongation is much more pronounced, especially along the height direction. Figure 11 links this macroscopic behavior to fracture morphology: compared with H-MDF, the MC-MDF specimens exhibit a more ductile fracture surface with denser dimples and a weaker tendency toward interfacial separation. Figure 12 further shows that MC-MDF promotes a higher recrystallized fraction and a more homogeneous grain structure during forging and solution treatment, thereby weakening the microstructural origin of directional ductility loss [27].

From the viewpoint of process optimization, the most effective route to suppress severe anisotropy is to break the continuity of one-directional streamline alignment and particle-string distribution by altering the strain path. Multidirectional forging and related multiaxial deformation routes are effective because they repeatedly reorient the principal stress state, fragment coarse particles, weaken texture sharpness, and promote more uniform recrystallization. Therefore, the goal is not merely to increase strength, but to transform a highly aligned one-dimensional fibrous structure into a more interwoven and orientation-tolerant three-dimensional microstructural network. In this sense, the reduction in anisotropy should be interpreted as a direct manifestation of improved flow-line homogeneity, rather than only as a conventional heat-treatment or grain-refinement effect [26,27,68].

A more quantitative comparison across representative studies further clarifies this point. In 7085 forgings processed by temperature-combination-controlled MDF, the elongation anisotropy index decreased from 14.0% to 8.6%, whereas the strength variation remained comparatively modest; in multidirectionally forged Al7075, the in-plane anisotropy of yield strength and tensile strength was reduced to about 1%; and in solution-forging-integrated 6082 components, improved flow-path coordination and microstructural uniformity were linked to more stable overall mechanical performance. These results collectively show that the most sensitive indicator of flow-line quality is often not the absolute strength level, but the reduction of directional scatter in plasticity and damage-tolerance-related behavior [23,26,27,74].

### 4.2. Fatigue Life Control and Notch Sensitivity

For forged aluminum alloy components, fatigue damage is controlled primarily by the near-surface region, because most fatigue cracks initiate at the free surface where cyclic tensile stress, geometric discontinuity, and microstructural heterogeneity interact. In this context, the morphology of flow lines, the presence of broken streamlines or lap-induced microcracks, and the surface roughness left by trimming or machining all act together in determining fatigue life. From a mechanistic viewpoint, surface roughness can be idealized as a population of micro-notches, and the Arola–Ramulu framework provides a useful basis for converting real surface topography into an equivalent stress-concentration problem [28,75]. In 6061-T6 aluminum alloy, Yang et al. further showed that the real surface profile can be simplified into a series of equivalent elliptic notches and then used for fatigue-life estimation, with the predicted life showing reasonable agreement with experiments [28]. Thus, for forged aluminum alloys, surface condition should not be treated as a secondary finishing issue; rather, it is one of the dominant fatigue-governing parameters.

This interpretation is particularly relevant when flow-line defects are present in the loaded region. Once exposed flow-line ends, folded interfaces, or particle-rich weak paths intersect the surface, they behave as highly effective crack-initiation sites, because the local notch effect is superimposed on pre-existing metallurgical discontinuities. In other words, geometric stress concentration and microstructural crack susceptibility no longer act independently. For forged 6082 aluminum alloy, Güngör and Edwards showed that small fatigue cracks initiated preferentially from coarse surface particles introduced during forging, and that the reduction in fatigue life was more strongly associated with these surface defects than with roughness alone [72]. A similar conclusion arises from multiaxial notch-fatigue work on 7050-T6 alloy, where crack initiation sites in notched specimens were successfully correlated with the first principal stress distribution at the notch surface, confirming that notch-root conditions dominate the earliest stage of fatigue damage accumulation [76]. Therefore, the fatigue sensitivity of forged aluminum alloys should be understood as the combined result of surface notch severity, streamline continuity, and defect-assisted local crack nucleation [72,76].

Importantly, the available evidence does not support a single universal scalar relationship between flow-line morphology and fatigue life. Instead, fatigue degradation becomes most severe when unfavorable streamline geometry coexists with a near-surface trigger such as machining damage, a notch root, a coarse particle cluster, or corrosion pitting. This explains why some studies identify roughness or machining integrity as the dominant factor, whereas others emphasize texture, particle distribution, or exposed flow-line ends: these variables act synergistically rather than competitively. For engineering assessment, the most defensible approach is therefore to evaluate flow-line continuity, surface integrity, and local stress concentration as a coupled reliability problem [25,28,62,63,64,65,72,75,76,77,78,79,80]. Recent studies on AA7010, AA7085, EN AW-2618A, and high-strength 6xxx alloys further reinforce this interpretation by showing that crack-path tortuosity, small-crack growth behavior, surface condition, and alloy-specific microstructural stability must be assessed together rather than as isolated variables [11,12,13,14,15,16].

The effect of temperature and loading spectrum further amplifies this sensitivity. Under constant-amplitude loading (CAL), high-to-low, and low-to-high spectrum loading, both AA6061-T6 and AA7075-T6 exhibit a pronounced reduction in fatigue life when the test temperature increases from room temperature to 250 °C; the total number of cycles to failure decreases by about 75–84%, showing that the fatigue resistance of heat-treatable aluminum alloys is highly temperature-sensitive [77]. However, even under such conditions, fatigue life is still strongly governed by the severity of the surface stress raisers rather than by nominal strength alone. This point is supported by microstructure-sensitive modeling of Al 7075-T6, which showed that roughness-induced notch depth is more detrimental to fatigue resistance than notch radius, because deeper surface valleys intensify the local fatigue driving force more effectively [78]. Accordingly, a continuous and contour-following flow-line network may be viewed as beneficial because it reduces abrupt surface interruption and promotes a more tortuous crack path, whereas broken streamlines and lap-related microcracks markedly increase the effective notch sensitivity of the component [77,78].

**Figure 13:**
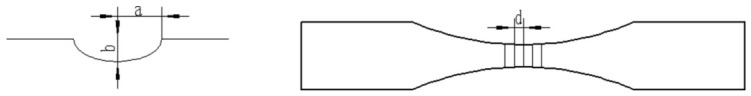
Geometry and dimensions of equivalent elliptic surface micro-notches used for fatigue-life analysis of 6061-T6 aluminum alloy based on surface roughness [67].

**Figure 14:**
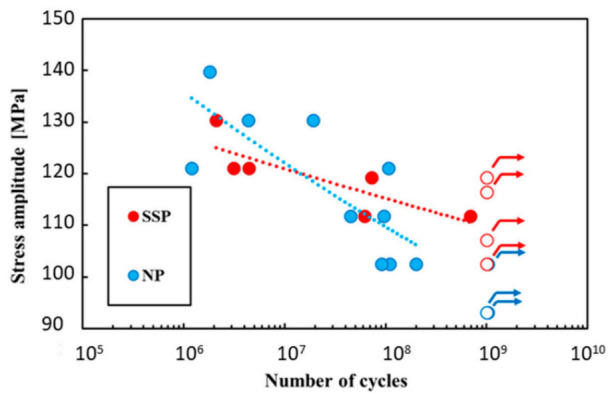
S–N curves of U-notched AW7075 aluminum alloy in the not-peened and severe-shot-peened states, showing the beneficial effect of shot peening in the very-high-cycle regime and the deterioration in shorter-life conditions [72].

Figure 13 provides the physical basis for the surface-roughness argument by showing the equivalent elliptic micro-notch geometry used in the roughness-based fatigue-life analysis of 6061-T6 aluminum alloy [28]. Figure 14 then provides direct evidence of notch-sensitive fatigue behavior in a high-strength aluminum alloy: the S–N curves of U-notched AW7075 specimens show that severe shot peening improves the fatigue limit in the very-high-cycle regime, but can reduce fatigue strength in the shorter-life regime because the beneficial compressive residual stress field is offset by surface damage and roughness-induced crack initiation [80]. Together, these figures show that fatigue-life control in forged aluminum alloys depends on both geometric surface condition and near-surface microstructural state.

From an engineering perspective, the control of fatigue life and notch sensitivity in forged aluminum alloys should therefore focus on preserving streamline continuity in the near-surface region, minimizing trimming- and machining-induced interruption of contour-following fibers, reducing roughness-derived micro-notch severity, and avoiding the superposition of geometric notches with particle-rich weak interfaces. For local strengthening methods such as shot peening, the benefit must also be balanced against the risk of introducing additional surface damage. In this sense, fatigue design for aluminum alloy forgings should not rely solely on static strength indices; it must explicitly incorporate surface integrity, micro-notch severity, and flow-line continuity as coupled variables in damage-tolerant design [28,72,75,76,77,78,79,80]. Critically, the current literature does not support a single universal relationship between streamline morphology and fatigue life. Some studies identify surface roughness or machining damage as the dominant factor, whereas others emphasize texture, particle clustering, or exposed flow-line ends. The most defensible conclusion is that fatigue sensitivity becomes severe when unfavorable flow-line geometry coexists with a near-surface crack-initiation site; accordingly, process design, trimming, and finishing should be evaluated as a coupled system rather than as isolated variables.

## 5. Digital Predictive Control and Process Optimization Strategies for Flow Lines

### 5.1. Theoretical Preform Design and Geometric Similarity Tracking

Preform design is the central problem in closed-die forging process optimization because it determines whether the material can fill the final die cavity progressively and smoothly, without generating severe local overloading, unstable flow redirection, or defect-prone free-surface inversion. In traditional industrial practice, blocker and preform geometries are still often determined by empirical rules, trial-and-error adjustment of fillet radii and draft angles, or simple geometric enlargement of the final shape. Although such approaches are practical, they are usually inefficient for complex components with deep ribs, local bosses, or highly nonuniform section transitions, because they cannot explicitly account for the actual strain path and velocity field during cavity filling [81,82,83].

A more rigorous route is provided by geometrical resemblance and backward material-flow tracing. In this framework, the target forging is first treated as the starting point, and the deformation history is reconstructed in reverse by tracking material points or boundary nodes backward through the FE-computed flow field. Rather than relying on a purely geometric guess, the reconstructed preform reflects the actual deformation physics, including friction, strain gradients, temperature effects, and die-contact evolution [84,85,86]. Earlier FEM-based backward-simulation studies already showed that inverse die-contact tracking can reduce flash and improve cavity filling in near-flashless forging [84], while sensitivity-based formulations were further developed to optimize die shape toward either net-shape accuracy or more uniform deformation [81,87]. These studies collectively indicate that preform design should be understood not as a static geometric matching problem, but as a flow-path control problem.

**Figure 15:**
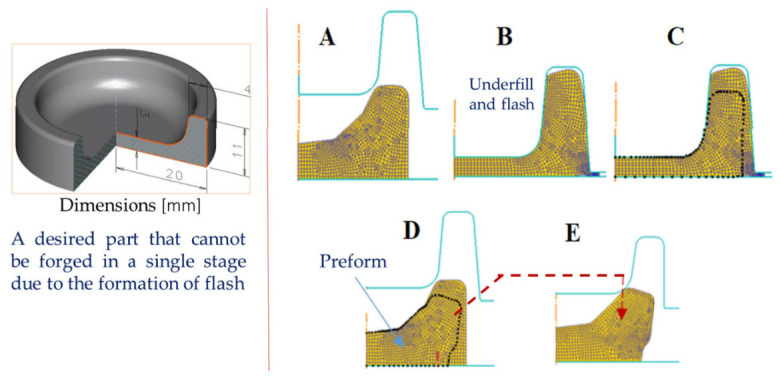
Illustration of point tracking in an FE model to obtain a preform [83]: (**A**) oversized initial geometry; (**B**) forged part with underfill/flash; (**C**) mapped points on the desired final geometry; (**D**) backtracked points on the initial billet; and (**E**) reconstructed preform shape.

**Figure 16:**
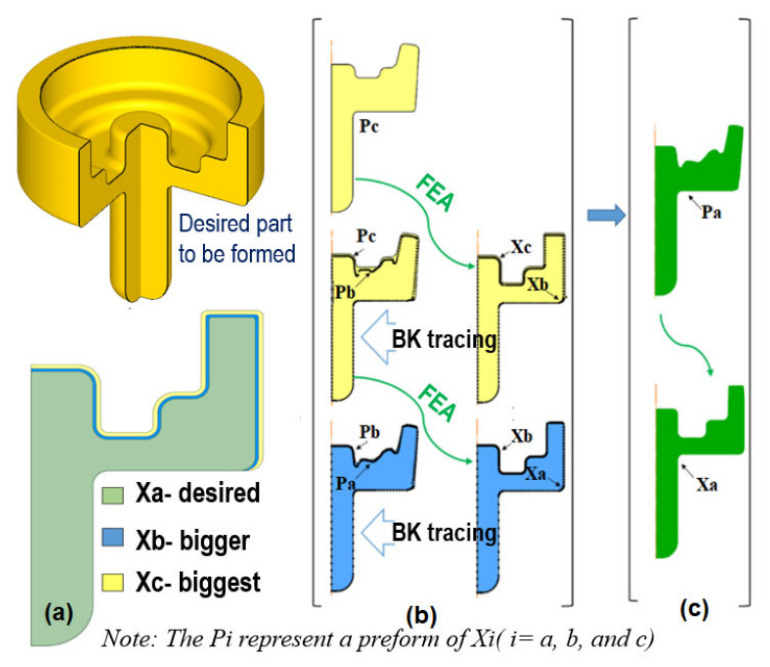
Geometrically resembling parts and iterative preform design [83]: (**a**) geometrically resembling intermediate shapes; (**b**) illustration of staged preform design for the target part; and (**c**) obtained preform and final part geometry.

The image evidence for this methodology should be introduced explicitly. Figure 15 shows the essential concept of point tracking in an FE model: a deliberately oversized initial geometry is first forged virtually, after which the boundary points of the desired final part are mapped and traced backward to reconstruct a physically informed preform [86]. This figure is important because it visualizes the transition from a defect-containing trial geometry to a backtracked and more suitable preform candidate. Figure 16 then extends this idea to the concept of geometrical resemblance and iterative convergence. As illustrated in the figure, a sequence of geometrically related intermediate shapes can be used to approach the final defect-free forging through a staged refinement of the preform. In a recent physics-informed study, this procedure was demonstrated in the form of a three-stage iterative scheme, showing that backtracking-based refinement can converge rapidly to preforms that reduce flash and forging load while maintaining satisfactory die filling [86]. Together, Figure 15 and Figure 16 clarify that geometric similarity tracking is not merely a geometric transformation, but an FE-informed inverse design strategy. Figure 15 illustrates the core logic of geometrical tracking in FE-based preform design: an oversized initial geometry is forged virtually, characteristic material points are traced backward, and the preform is iteratively corrected until cavity filling and strain distribution become more uniform.

On this basis, recent developments have moved toward data-driven and AI-assisted preform design. Convolutional neural network (CNN)-based methods extract the geometric features of forged parts directly from voxelized shape data and learn their relationship to appropriate preform geometries through training datasets generated by FE simulations [88,89]. Compared with classical iterative methods, such models can provide multiple preform candidates rapidly and reduce dependence on designer intuition. More recent studies have further combined generative AI architectures with surrogate models, allowing preform candidates to be explored under multi-objective criteria such as flash reduction, load minimization, and defect avoidance [90]. These methods do not eliminate the need for FE verification; rather, they shift FE analysis from a primary search tool to a validation and ranking tool for AI-generated candidates.

**Figure 17:**
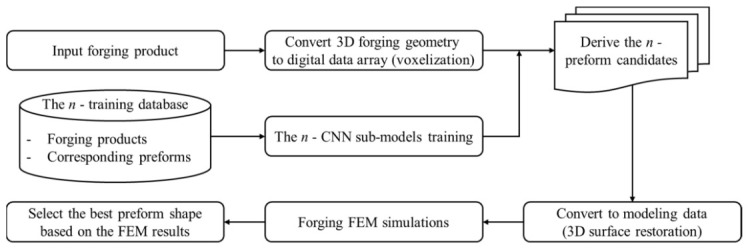
Flowchart for the CNN-based preform design methodology, including voxelization of the target forging geometry, generation of candidate preforms by trained CNN sub-models, and FEM-based evaluation of the derived designs [80].

This transition is best summarized by Figure 17, which presents the workflow of a CNN-based preform design methodology [88]. The figure shows that the target forging geometry is first converted into a three-dimensional data array, after which trained CNN sub-models generate several candidate preform geometries; these are then evaluated by FEM, and the most suitable design is selected according to load reduction and defect-avoidance criteria. In other words, the current trend in preform design is evolving from empirical drafting, to physics-based backward tracing, and further to hybrid intelligent design, in which FE-derived material-flow knowledge and machine-learning-based geometric inference are integrated. For aluminum alloy forgings, this evolution is particularly important because precise preform control can directly reduce the probability of folding, flow-through, and local streamline interruption in subsequent forming stages [81,82,83,84,85,86,87,88,89,90].

### 5.2. Multiphysics Mathematical Modeling and Folding Index Evaluation

To reproduce the deformation behavior of aluminum alloys under complex forging conditions, modern simulation frameworks increasingly rely on multiphysics and multiscale mathematical modeling. At the process scale, thermo-mechanical FE models couple heat transfer, frictional contact, plastic flow, and evolving strain-rate fields to predict temperature, strain, and stress distributions throughout the billet. In current practice, the constitutive response is commonly introduced through Arrhenius-type flow laws or other user-defined rheological equations, while microstructure-evolution subroutines are further embedded to capture dynamic recrystallization and grain-size variation during deformation [91,92,93]. At a higher thermodynamic level, CALPHAD-coupled kinetic models provide quantitative descriptions of precipitation and phase evolution in multicomponent aluminum alloys, thereby offering physically informed inputs for integrated computation frameworks rather than purely empirical constitutive fitting [94]. In this sense, multiphysics modeling in forging should not be viewed simply as “load and shape prediction”, but as a hierarchical coupling of flow behavior, thermal history, and microstructural evolution.

A related and more relevant modeling direction for the present review concerns the coupled prediction of local temperature, friction, damage initiation, and void evolution under large-strain forging conditions [95,96,97,98]. In aluminum alloy die forging, local tensile hydrostatic stress, severe strain gradients, and thermal non-uniformity can promote internal discontinuities or weak-path development even when the nominal macroscopic filling sequence appears acceptable. Therefore, the most useful extensions of multiphysics modeling are those that remain forging-centered and explicitly connect thermal-mechanical fields to flow-line continuity, defect susceptibility, and microstructural heterogeneity.

Within forging-specific optimization, one of the most useful quantitative tools is the folding objective function, which can also be interpreted as a generalized folding index. Instead of depending on a single geometric length or cavity dimension, this method evaluates folding tendency through the time-averaged spatial integration of the equivalent plastic strain rate over the free surface, thereby converting free-surface instability into a dimensionless optimization target [91]. This is an important conceptual advance, because it shifts fold prediction from post hoc geometric judgment to field-based mathematical evaluation. Once expressed in this form, the folding index can be incorporated directly into FE-driven optimization frameworks and used together with metamodeling tools, such as response surface methodology, grey relational analysis, and other multi-objective strategies, to identify parameter combinations that minimize fold tendency while controlling load, stress, and damage [91,92,93,94,95,96,97,98,99]. In recent process-parameter studies, orthogonal design, ANOVA, and response-surface-type optimization have been successfully used to quantify the influence of billet temperature, punch speed, and friction coefficient on fold-related responses, showing that friction is often the most sensitive variable in fold formation [99,100].

**Figure 18:**
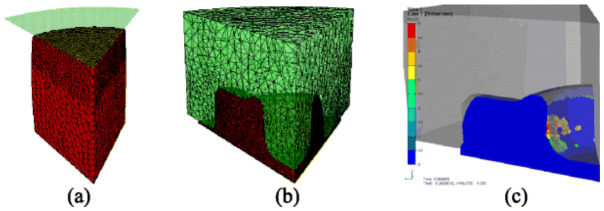
FE results illustrating the formation of folding in a two-step forging process [84]: (**a**) FE model after upsetting; (**b**) FE model after forging; and (**c**) localized free-surface deformation associated with folding. The depicted quantity is the equivalent plastic strain rate at the free surface.

**Figure 19:**
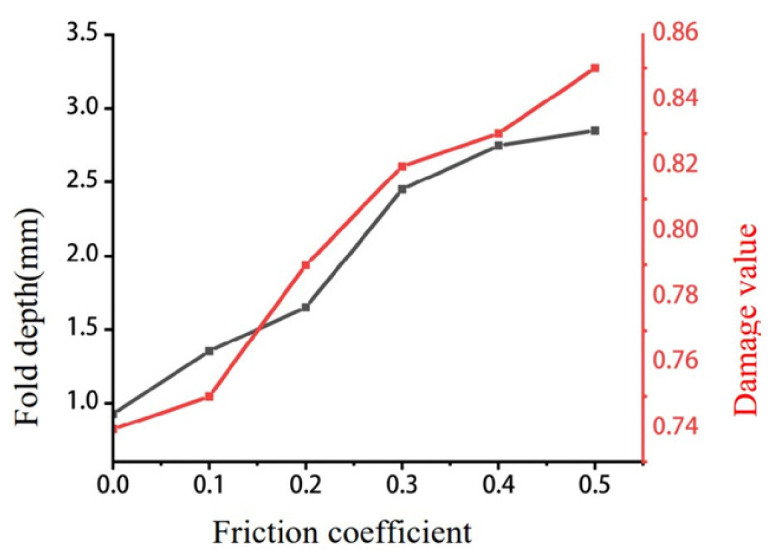
Further shows that fold depth and damage value vary systematically with the friction coefficient, demonstrating that fold-related quantitative indicators can be used directly in parameter screening and process optimization [93].

Figure 18 provides the mathematical and physical basis for the folding-index concept: the FE model identifies a localized concentration of equivalent plastic strain rate at the free surface during forging, and the folded region appears where the free-surface deformation becomes unstable [91]. Figure 19 further shows that fold depth and damage value vary systematically with the friction coefficient, demonstrating that fold-related quantitative indicators can be used directly in parameter screening and process optimization [100]. Together, these figures show that modern fold control is increasingly based on field variables, mathematical indicators, and optimization-driven process design.

Overall, the development of multiphysics mathematical modeling has transformed defect control in forging from a trial-and-error practice into a predictive and optimizable design problem. Thermo-mechanical FE analysis provides the process-scale framework, CALPHAD and kinetic models strengthen the thermodynamic and microstructural basis, and folding-index-type criteria connect free-surface instability directly to quantitative optimization. For aluminum alloy forgings, this integration is particularly valuable because it enables the simultaneous control of forming load, flow-line stability, microstructural homogeneity, and defect sensitivity within a unified digital-design framework [91,92,93,94,95,96,97,98,99,100].

### 5.3. Frictional Heat Management and Isothermal Forging Practice

At the practical process level, the final stabilization of flow trajectories depends strongly on the control of interfacial friction and heat transfer. For aluminum alloys, this issue is particularly critical because their relatively high thermal conductivity and narrow workable temperature window make them sensitive to die chilling, local flow retardation, and adhesion to the tool surface. Under conventional hot forging conditions, severe temperature gradients may develop rapidly between the billet core and the surface layer in contact with the die, which promotes non-uniform deformation, increases local flow resistance, and may aggravate defects such as underfilling, streamline interruption, or surface tearing. In this context, isothermal or near-isothermal precision forging has become an important route for producing complex aluminum alloy components with improved dimensional accuracy and more favorable contour-following flow lines [101,102,103,104]. In classical isothermal precision forging of a cylindrical 7075 aluminum-alloy housing, for example, the dies were preheated to 460 ± 10 °C and maintained near the billet temperature during forging, while a graphite–water lubricant was applied at the interface; under these conditions, the forging could be filled successfully with little or no subsequent machining [95]. A similar process logic was reported for fully enclosed isothermal forging of an aluminum-alloy rotor, where temperature-controlled dies and billet heating enabled the twisted blades to be formed close to final contour while preserving streamline conformity and reducing machining time [103].

The key advantage of isothermal forging is that it suppresses the most harmful thermal gradients at the die–workpiece interface. When die chilling is reduced, the surface layer is less likely to harden prematurely, and the metal can flow more uniformly into thin ribs, deep cavities, or combined extrusion features. This is one reason why isothermal precision forging is repeatedly associated with improved die filling, lower forging pressure, and better flow-line distribution in complex aluminum forgings [101,102,103,104]. In the 7A09 rotating-disk study, digital design combined with processing-map-guided isothermal forging at 430 °C and a low ram speed enabled the manufacture of a high-quality rotating disk with good agreement between simulation and experiment, indicating that stable thermal control is a prerequisite for defect-free filling and predictable streamline evolution [98]. Accordingly, isothermal forging should be viewed not merely as die heating, but as a process strategy for simultaneously reducing surface flow resistance, stabilizing the velocity field, and preserving contour-following deformation paths [101,103,104].

Interfacial lubrication is the second essential pillar of this strategy. Even when the thermal field is well controlled, aluminum alloys exhibit a strong tendency toward sticking, galling, and die pick-up at elevated temperature. The severity of this behavior depends on temperature, pressure, sliding speed, and the integrity of the lubricant film or coating [105,106,107,108,109]. For 7075 aluminum alloy, inverse-analysis-based hot-stamping experiments showed that lubrication markedly lowers the forming load and improves the surface quality of formed parts; among the tested lubricants, molybdenum disulfide gave the best overall lubrication effect, whereas unlubricated parts suffered severe scratches or even cracking [106]. A broader tribological study on a 7000-series aluminum sheet likewise showed that lubricant performance and PVD die coatings must be evaluated together, from coupon-level friction tests to component-level forming, because lubricant breakdown at elevated temperature directly affects material transfer to the die and hence final part quality [107]. In addition, unlubricated sliding tests on 7075 aluminum alloy revealed that the dominant friction mechanism changes from ploughing at lower temperatures to adhesive peeling at temperatures above 300 °C, illustrating why frictional heat and galling become especially harmful in hot aluminum forming [108].

**Figure 20:**
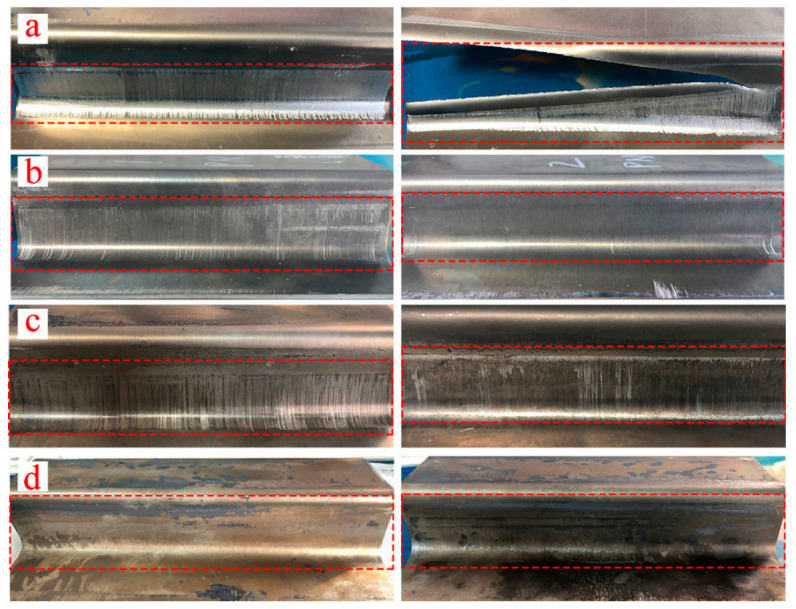
U-shaped parts of 7075 aluminum formed under different lubrication conditions [99]: (**a**) no lubricant; (**b**) boron nitride lubricant; (**c**) graphite lubricant; and (**d**) molybdenum disulfide lubricant.

**Figure 21:**
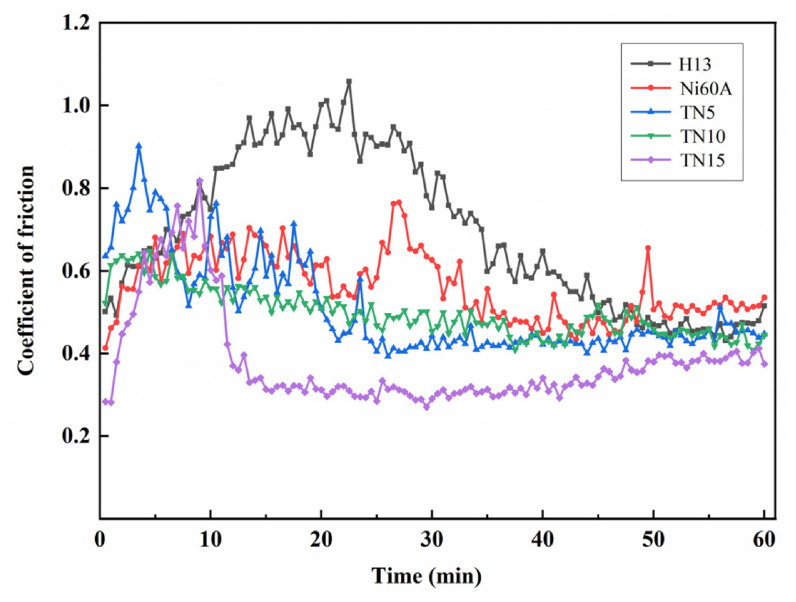
Further shows that TiB_2_-reinforced Ni-based coatings reduce the friction coefficient and suppress severe adhesion during high-temperature sliding against Al-7075, demonstrating the value of die-surface engineering for frictional heat management [98].

Figure 20 provides direct process evidence that lubrication quality strongly affects the forming outcome. U-shaped 7075 aluminum parts formed without lubricant exhibited severe scratching or cracking, whereas the specimens formed with molybdenum disulfide lubricant showed the best surface quality among the tested conditions [106]. Figure 21 complements this observation by showing that Ti-reinforced Ni-based coatings reduce the friction coefficient and suppress severe aluminum adhesion during high-temperature sliding against Al-7075 [105]. Together, these results indicate that frictional heat management in aluminum forging depends on the combined control of temperature matching, lubricant stability, and die-surface engineering.

More advanced lubrication strategies are now emerging from high-temperature tribology and die-surface engineering. In hot forging of lubricated aluminum alloy, Lee et al. introduced the concept of critical surface strain, showing that the effective Coulomb friction coefficient can change abruptly once the surface strain reaches a level at which the solid lubricating film is disrupted [109]. This result is particularly relevant for flow-line control because it links lubricant failure directly to local surface deformation, rather than treating friction as a fixed material constant. At the same time, self-lubricating die coatings and nanoparticle-based high-temperature lubricants are being actively explored. TiB_2_-reinforced Ni-based coatings were shown to reduce adhesion and generate boron-rich lubricating products during sliding against Al-7075 [105], while WS_2_ nanopowders have been demonstrated to maintain very low friction at elevated temperature because of the easy shear of the (002) basal plane and the formation of oriented lubricating films [109]. These advances suggest that the future of isothermal aluminum forging lies in coupling controlled thermal fields with adaptive high-temperature interfacial lubrication, rather than relying on temperature control alone.

Overall, frictional heat management and isothermal forging practice should be regarded as the final operational safeguard for flow-line optimization. By minimizing die chilling, suppressing sticking, and stabilizing interfacial sliding, these methods reduce the likelihood of local flow stagnation, streamline interruption, and defect accumulation in complex cavities. In engineering terms, their value lies not only in lowering the forging load, but also in enabling near-net-shape forming with improved surface quality, more stable flow trajectories, and reduced downstream machining demand [101,102,103,104,105,106,107,108,109].

Before moving to the final conclusions, three research gaps deserve emphasis. First, most current evidence is post-mortem rather than in situ, so quantitative validation of transient flow-line evolution remains limited. Second, defect criteria are still heterogeneous across studies, which complicates cross-process comparison and industrial transfer. Third, digital optimization workflows are improving rapidly, but closed-loop integration between FE simulation, sensing, and metallographic validation is still rare in production forging. These issues explain why terminology, evidence level, and control metrics remain uneven across the literature. To synthesize the preceding discussion, Table 3 compares the principal process-optimization strategies for flow-trajectory control, emphasizing their major benefits, practical limitations, and preferred deployment stages. In addition, the certainty level is not uniform across the topics reviewed here: folding, anisotropy, and fatigue-notch interactions are supported by comparatively mature forging evidence, whereas vortex-like instability, some cross-process analogies, and several microstructure-to-life inferences remain more interpretive. This distinction should be kept explicit when translating current literature into process-design rules.

As shown in Table 3, no single strategy can independently guarantee stable flow-line evolution; the most robust route combines preform design, multiphysics simulation, and thermal-tribological control within an integrated optimization framework.

## 6. Conclusions and Outlook

Flow-line evolution is a fundamental yet still insufficiently quantified issue in aluminum alloy forging. As discussed throughout this review, flow lines are not merely metallographic traces left by plastic deformation, but direct manifestations of material transport, thermomechanical compatibility, microstructural evolution, defect formation, and subsequent service behavior. Their continuity, orientation, and spatial distribution provide a critical link between forging-process design and component reliability.

(1) Flow trajectories in aluminum alloy forging are governed by the coupled effects of constitutive flow behavior, dynamic softening, second-phase particle redistribution, thermal gradients, frictional conditions, and strain-path evolution. Stable and contour-following flow lines are generally associated with coordinated material transport and satisfactory die filling, whereas disturbed or discontinuous flow trajectories indicate local deformation incompatibility and a high tendency toward defect formation.

(2) Abnormal flow-line evolution is the essential origin of several typical forging defects, including folding, flow-through, vortex-like instability, and exposed or broken streamlines. Although these defects differ in morphology and scale, they share a common physical basis, namely the mismatch of velocity, temperature, deformation resistance, and interfacial constraint between adjacent regions. Therefore, flow-line regulation should be considered a primary defect-prevention strategy, rather than a secondary metallographic concern after forging.

(3) Flow trajectories have a decisive influence on the mechanical performance and long-term durability of forged aluminum alloy components. Continuous and properly oriented flow lines can improve deformation compatibility, retard crack propagation, and enhance fatigue resistance and structural integrity. In contrast, interrupted, disordered, or exposed flow lines intensify plastic anisotropy, notch sensitivity, and environmentally assisted damage. In many practical cases, the most severe consequence of abnormal flow-line evolution is not a large reduction in strength, but a pronounced deterioration in ductility, damage tolerance, and service reliability.

Future work should establish a more quantitative and unified framework for flow-line control in aluminum alloy forging. Priority areas include multiscale predictive models linking flow-line evolution with recrystallization, particle fragmentation, residual stress, crack initiation, and environmental degradation; advanced in situ and three-dimensional characterization methods for reconstructing transient flow paths; and AI-assisted design strategies that integrate preform optimization, multiphysics simulation, and defect evaluation within a digitally closed-loop process chain. At the same time, future predictive frameworks should distinguish clearly between phenomena already supported by direct forging evidence and those inferred from adjacent processing routes, so that the hierarchy of confidence remains explicit in both academic analysis and industrial deployment.

## Figures and Tables

**Table 1:** Terminology and scope framework adopted in this review.

Term	Definition Used in This Review	Typical Manifestation in Forging	Scope Note
Flow trajectories	Overall evolving material-flow path during deformation	Bulk cavity-filling route and local redirection of metal flow	Used as the umbrella concept throughout the review
Flow lines	Observable metallographic or macrostructural manifestation of prior flow	Fiber-like bands, streamline patterns, etched flow-line contours	Treated as evidence of previous flow history rather than the whole process itself
Flow-line discontinuity	Loss of continuity, abrupt turning, or local penetration of aligned flow paths	Broken streamlines, exposed ends, internal weak paths	Considered the immediate precursor to several forging defects
Folding/lap	Surface layer self-contact caused by incompatible local surface kinematics	Overlap near fillets, ribs, webs, or flash regions	Discussed as the most mature defect class in current forging literature
Flow-through defect	Internal streamline penetration or abnormal through-flow between adjacent regions	Band-like internal discontinuity and property mismatch	Interpreted as a combined kinematic and metallurgical problem

**Table 2:** Comparative summary of abnormal flow-trajectory-induced defects in aluminum alloy forging.

Defect Type	Primary Trigger	Main Metallurgical/Mechanical Consequence	Representative Prevention Strategy
Folding/lap	Abrupt surface-flow redirection, local volume excess, high friction, sharp die transitions	Unbonded overlap, stress concentration, crack initiation at near-surface weak interfaces	Preform optimization, fillet-radius improvement, friction control, temperature homogenization
Flow-through defect	Severe resistance gradient and internal streamline penetration between adjacent zones	Internal continuity loss, heterogeneous strain/texture, local weak path for fracture	Balanced sectional reduction, coordinated material distribution, forging-sequence redesign
Vortex-like instability	High local velocity, sudden cavity expansion, strong momentum redistribution	Potential entrainment, local non-uniformity, uncertain near-surface quality risk	Limit high-speed impingement zones and verify with forging-centered simulations
Exposed flow lines	Unfavorable metal discharge, improper trimming or machining, streamline truncation at the surface	Corrosion-fatigue sensitivity and reduced crack-arrest capability	Trim allowance control, contour-aligned flow design, surface integrity management

**Table 3:** Comparison of current process-optimization strategies for flow-trajectory control.

Strategy	Principal Benefit	Key Limitation	Best Use Stage
Geometrical preform design	Improves filling sequence and reduces abrupt streamline turning	Highly dependent on designer experience if used alone	Early process planning
FE-based similarity tracking/backtracking	Provides physics-based correction of local flow-path mismatch	Requires reliable constitutive data and repeated simulation updates	Preform and die refinement
Folding-index or defect objective functions	Transforms qualitative defect risk into an optimizable quantitative metric	Indicator transferability across alloys and geometries is still limited	Parameter optimization and sensitivity analysis
Frictional heat management and isothermal forging	Stabilizes near-surface flow and suppresses chilling-induced non-uniformity	Tooling cost and process window can be restrictive	Shop-floor execution and high-value precision forging
Data-driven/AI-assisted design	Accelerates exploration of complex geometry-process relationships	Model transparency and validation remain insufficient for many industrial cases	Rapid iteration after a validated physics baseline

## Data Availability

No new data were created or analyzed in this study. Data sharing is not applicable to this article.

## Abbreviations

Abbreviation	Definition
CAL	Constant-amplitude loading
CALPHAD	Calculation of phase diagrams
CDRX	Continuous dynamic recrystallization
CNN	Convolutional neural network
DDRX	Discontinuous dynamic recrystallization
DRV	Dynamic recovery
DRX	Dynamic recrystallization
EBSD	Electron backscatter diffraction
FE/FEM	Finite element/finite element method
GOS	Grain orientation spread
HAGB	High-angle grain boundary
IPF	Inverse pole figure
KAM	Kernel average misorientation
LAGB	Low-angle grain boundary
MAD	Misorientation angle distribution
MC-MDF	Temperature-combination-controlled multidirectional forging
MDF	Multidirectional forging
PSN	Particle-stimulated nucleation
SCC	Stress corrosion cracking
SEM	Scanning electron microscopy
TEM	Transmission electron microscopy
UTS	Ultimate tensile strength
YS	Yield strength
